# A Novel Identification Methodology for the Coordinate Relationship between a 3D Vision System and a Legged Robot

**DOI:** 10.3390/s150409519

**Published:** 2015-04-22

**Authors:** Xun Chai, Feng Gao, Yang Pan, Chenkun Qi, Yilin Xu

**Affiliations:** State Key Laboratory of Mechanical System and Vibration, Shanghai Jiao Tong University, Shanghai 200240, China; E-Mails: chaixun@sjtu.edu.cn (X.C.); py0330@gmail.com (Y.P.); chenkqi@sjtu.edu.cn (C.Q.); xuyilin@sjtu.edu.cn (Y.X.)

**Keywords:** coordinate identification methodology, 3D vision system, hexapod robot

## Abstract

Coordinate identification between vision systems and robots is quite a challenging issue in the field of intelligent robotic applications, involving steps such as perceiving the immediate environment, building the terrain map and planning the locomotion automatically. It is now well established that current identification methods have non-negligible limitations such as a difficult feature matching, the requirement of external tools and the intervention of multiple people. In this paper, we propose a novel methodology to identify the geometric parameters of 3D vision systems mounted on robots without involving other people or additional equipment. In particular, our method focuses on legged robots which have complex body structures and excellent locomotion ability compared to their wheeled/tracked counterparts. The parameters can be identified only by moving robots on a relatively flat ground. Concretely, an estimation approach is provided to calculate the ground plane. In addition, the relationship between the robot and the ground is modeled. The parameters are obtained by formulating the identification problem as an optimization problem. The methodology is integrated on a legged robot called “Octopus”, which can traverse through rough terrains with high stability after obtaining the identification parameters of its mounted vision system using the proposed method. Diverse experiments in different environments demonstrate our novel method is accurate and robust.

## 1. Introduction

The use of sensors, especially vision sensors and force sensors, which provide robots with their sensing ability, plays an important role in the intelligent robotic field. Legged robots, after having a good knowledge of the spot environment, can previously select a safe path and a set of appropriate footholds, then plan the feet and body locomotion effectively in order to traverse rough terrains automatically with high stability, velocity and low energy consumption. There have been some related examples in recent years. HyQ [1] can trot on uneven ground based on its vision-enhanced reactive locomotion control scheme, by using an IMU and a camera. Messor [2] uses a Kinect to classify various terrains in order to achieve automatic walking on different terrains. The DLR Crawler [3] can navigate in unknown rough terrain using a stereo camera. Little Dog [4] uses a stereo camera and the ICP algorithm to build the terrain model. Messor [5] uses a laser range finder to build the elevation map of rough terrains, and chooses appropriate foothold points based on the elevation map. Planetary Exploration Rover [6] builds a map model with a LIDAR sensor based on the objects’ distance and plans an optimized path. AMOS II [7] uses a 2D laser range finder to detect the distance to obstacles and gaps in front of the robot, and it also can classify terrains based on the detected data. A humanoid robot [8] uses a 3D TOF camera and a webcam camera to build a digital map, and after that plans a collision avoiding path. Another humanoid robot [9] can walk along a collision avoiding path based on fuzzy logic theory with the help of a webcam camera. The RHEX robot [10] is able to achieve reliable 3D sensing and locomotion planning with a stereo camera and an IMU mounted on it.

In robotics, if a vision sensor is mounted on the robot, its pose with respect to the robot frame must be known, otherwise the vision information can’t be used by the robot. However, only a few works describe how to compute it. In related fields, problems of extrinsic calibration of two or more vision sensors have been studied extensively. Herrera [11] proposed an algorithm that could calibrate the intrinsic parameters and the relative position of a color camera and a depth camera at the same time. Li, Liu *et al*. [12] used the straight line features to identify the extrinsic parameters of a camera and a LRF. Guo *et al*. [13] solved the identification problem of a LRF and a camera by using the least squares method twice. Geiger *et al*. [14] presented a method which can automatically identify the extrinsic parameters of a camera and a range sensor using one shot. Pandey and McBride [15] successfully performed an automatic targetless extrinsic calibration of a LRF and a camera by maximizing the mutual information. Zhang and Robert [16] proposed a theoretic algorithm calibrating extrinsic parameters of a camera and a LRF by using a chessboard, and they also verified the theory by experiments. Huang *et al.* [17] calibrated the extrinsic parameters of a multi-beam LIDAR system by using V-shaped planes and infrared images. Fernández-Moral *et al*. [18] presented a method for identifying the extrinsic parameters of a set of range finders by finding and matching planes in 5 s. Kwak [19] used a V-shaped plane as the target to calibrate the extrinsic parameters of a LIDAR and a camera by minimizing the distance between corresponding features. By using a spherical mirror, Agrawal [20] could achieve extrinsic calibration parameters of a camera without a direct view. When two vision sensors don’t have overlapping detection regions, Lébraly *et al*. [21] could obtain the extrinsic calibration parameters using a planar mirror. By using a mirror to observe the environment from different viewing angles, Hesch *et al*. [22] determined the extrinsic identification parameters of a camera and other fixed frames. Zhou [23] proposed a solution for the extrinsic calibration of a 2D LIDAR and a camera using three plane-line correspondences. Kelly [24] used GPS measurements to establish the scale of both the scene and the stereo baseline, which could be used to achieve simultaneous mapping.

A more related kind of work is the coordinate identification between a vision system and manipulators. Wang [25] proposed three methods to identify the coordinate systems of manipulators and a vision sensor, then compared them by simulations and experiments. Strobl [26] proposed an optimized robot hand-eye calibration method. Dornaika and Horaud [27] presented two solutions to perform the robot-world and the hand-eye calibration simultaneously, one was a closed-formed method which used the quaternion algebra and a positive quadratic error function, the other one was based on a nonlinear constrained minimization, they found that the nonlinear optimization method was more stable with respect to noises and measurements errors. Wong wilai [28] used a Softkinetic Depthsense, which could acquire distance images directly, to calibrate an eye-in-hand system.

Few papers and researches involve identifying the coordinate relationship between the vision system and legged robots. The most similar and recent work to our own is that of Hoepflinger [29], which calibrated the pose of a RGB-D camera with respect to a legged robot. Their method needed to recognize the foot position in the camera coordinate system based on the assumption that the robot’s foot has a specific color and shape. Then identification parameters can be obtained by comparing the foot position in different coordinate systems, the camera frame and the robot frame. Our research target is the same with theirs, while the solution is totally different.

Existing methods to identify extrinsic parameters of the vision sensor suffer from several disadvantages, such as a difficult featuring matching or recognition, requirement for external equipment and the involvement of human interventions. Current identification approaches are often elaborate procedures. Moreover, work has seldom been done for the pose identification of the vision sensor mounted on legged robots. To overcome limitations of the existing methods and supplement relevant study in legged robots, in this paper we propose a novel coordinate identification methodology for a 3D vision system mounted on a legged robot without involving other people or additional equipment. This paper makes the following contributions:

1. A novel coordinate identification methodology for a 3D vision system of a legged robot is proposed, which needs no additional equipment or human inventions.
2. We use the ground as the reference target, which makes it possible for our methodology to be widely used. At the same time, an estimation approach is introduced based on the optimization and statistical methods to calculate the ground plane accurately.
3. The relationship between the legged robot and the ground is modeled, which can be used to precisely obtain the pose of the legged robot with respect to the ground.
4. We integrate the proposed methodology on “Octopus”, which can traverse rough terrains after obtaining the identification parameters. Various experiments are carried out to validate the accuracy and robust of the method.

The remainder of this paper is organized as follows: Section 2 provides a brief introduction to the robot system. Section 3 describes the problem formulation and the definition of coordinate systems. Section 4 presents the modeling and the method in detail. Section 5 describes the experiments and discusses the error and robust analysis results. Section 6 summarizes and concludes the paper.

## 2. System Description

**Figure 1:**
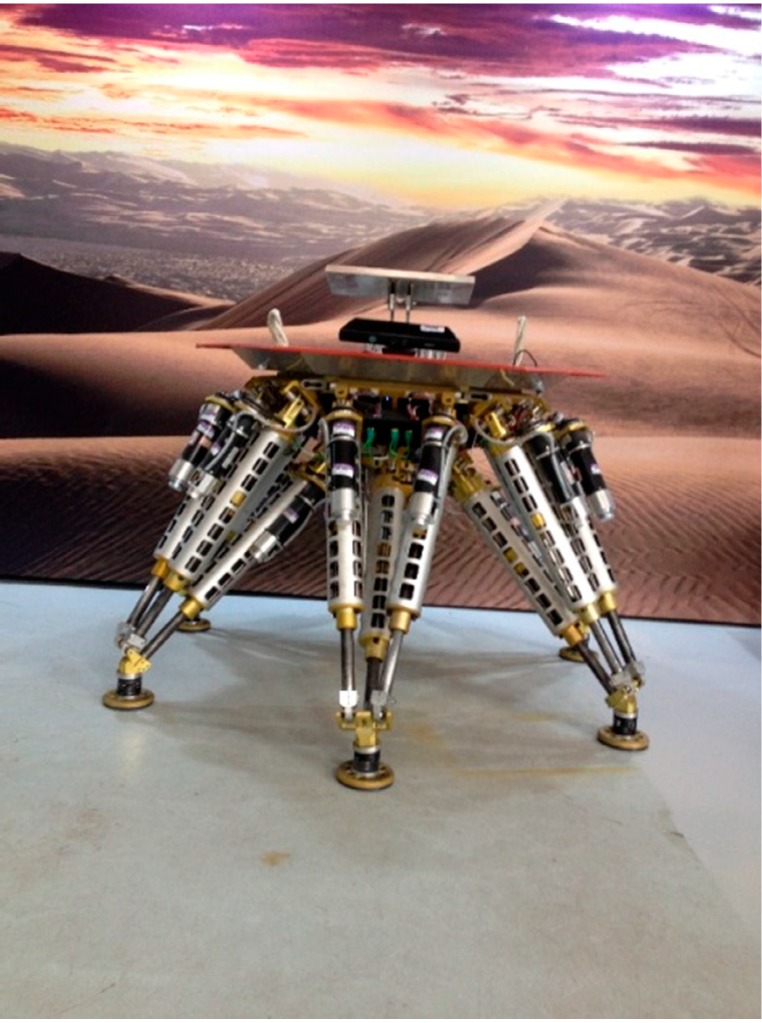
The legged robot “Octopus”.

The legged robot is called “Octopus” [30,31], which has a hexagonal body with six identical legs arranged in a diagonally symmetrical way around its body as shown in Figure 1. The robot is a six DOFS moving platform that integrates walking and manipulating. A vision system is necessary for building a terrain map, and its mounting position and orientation with respect to the robot frame, which is essential for locomotion planning, need to be acquired.

**Figure 2:**
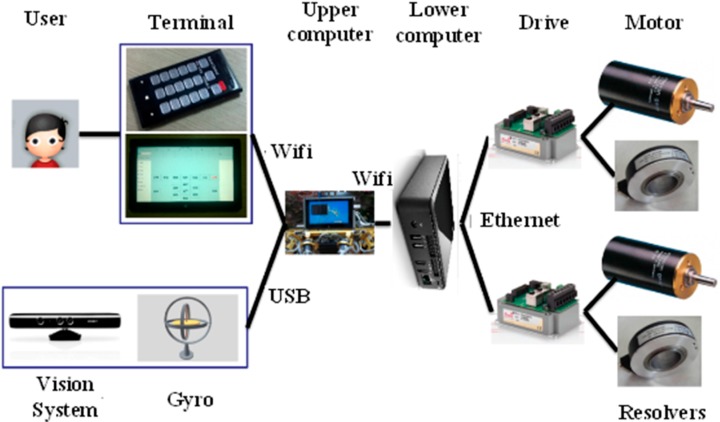
The control architecture of the robot.

Figure 2 shows the control architecture of the robot. Users send commands to the upper computer via a control terminal, which can be a smart phone or a pad and communicates with the upper computer via Wi-Fi. The sensor system contains a 3D vision sensor, a gyro, a compass and an accelerometer. The 3D vision sensor detects the terrain in front of the robot and provides the 3D coordinate data. The 3D vision sensor connects with the upper computer via USB. The compass helps the robot navigate in the right direction in outdoor environments. The gyro and the accelerometer can measure the inclination, the angle velocity and the linear acceleration of the robot. The upper computer is a super notebook, which receives and processes useful data from the sensor system. The upper computer sends instructions to the lower computer via Wi-Fi too. The Wi-Fi networking is created by the upper computer. The lower computer runs a real-time Linux OS. The lower computer analyzes messages sent by the upper computer, then plans locomotion and sends planned data to drivers via Ethernet at run time. Drivers provide current to motors, and servo control motors using the feedback data from resolvers.

**Figure 3:**
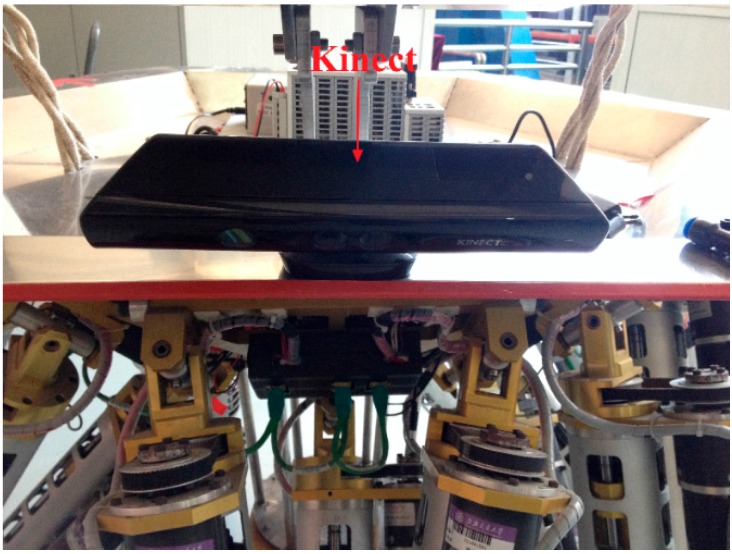
The 3D vision sensor.

The current work we are doing is try to make the robot walk and operate automatically in unknown environments with the help of the 3D vision sensor. Automatic locomotion planning needs the 3D coordinates of the surroundings, which can be transferred from depth images captured by the 3D vision sensor. Common laser range finders can only measure distances to objects that are located in the laser line of sight, while the 3D vision sensor can measure all the distances to objects in the range of the detection region, which is the reason why we choose a 3D vision sensor. The 3D vision sensor we use is a Kinect (as Figure 3 shows), which integrates multiple kinds of useful sensors, consisting of a RGB camera, an infrared emitter and camera, and four microphones. The RGB camera can capture 2D RGB images, the infrared emitter and camera constitute a 3D depth sensor which can measure the distance. Speech recognition and sound source localization can be achieved by processing voice messages obtained by the four microphones at the same time.

**Figure 4:**
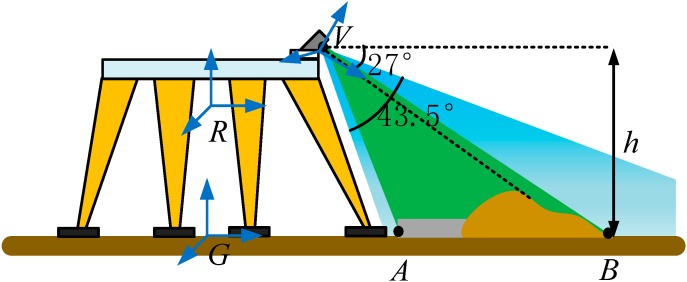
Installation schematic diagram of the 3D vision system.

Equipped with the 3D vision sensor, the robot can see objects from 0.8 m to 4 m and has a 57.5° horizontal vision angle and 43.5° vertical vision angle. The range from 1.2 m to 3.5 m is a sweet spot, in which the measuring precision can reach millimeter level [32,33]. Additionally, a small motor inside the 3D vision sensor allows it to tilt up and down from −27° to 27°. The 3D vision sensor is installed at the top of the robot as Figure 4 shows. The motor is driven to make the 3D vision sensor tilt down in order to ensure it can detect the terrain in front. The blue area is the region that the 3D vision sensor can detect, and the green area is the sweet spot. The height of the 3D vision sensor, denoted by *h*, is about 1 m. The short border VA of the green area is about 1.2 m and the long border VB is about 3.5 m through geometric calculations. We can make sure the depth data in the green area have a higher precision.

## 3. Problem Formulation and Definition of Coordinate Systems

**Figure 5:**
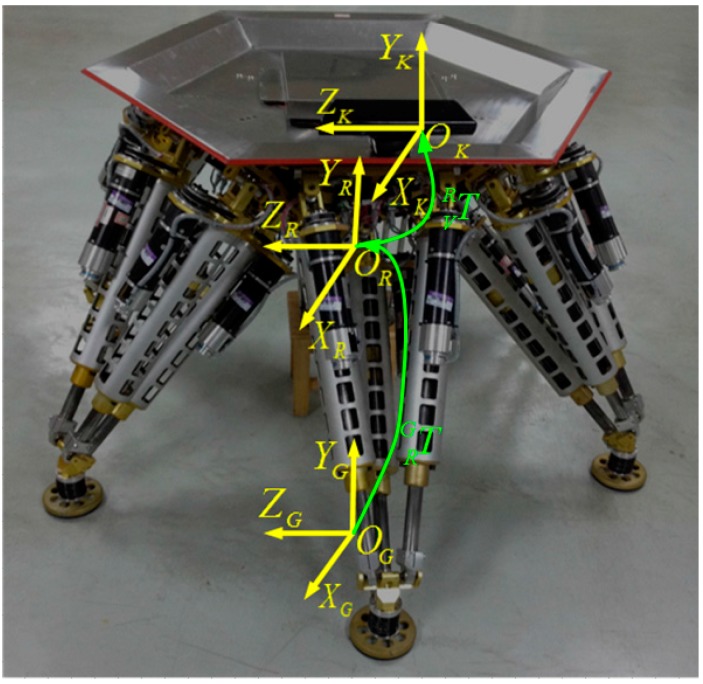
Definition of coordinate systems.

As mentioned above, it is very important to know the exact relationship between the 3D vision sensor coordinate system and the robot coordinate system. In other words, the mounting position and orientation of the 3D vision sensor must be identified. In order to express this simply, we us G-CS as short notation for the ground coordinate system, R-CS is short for the robot coordinate system, and V-CS is short for the 3D vision sensor coordinate system. As Figure 5 shows, the G-CS is represented by $O_{G}X_{G}Y_{G}Z_{G}$, which is used as the reference target with respect to the V-CS represented by $O_{V}X_{V}Y_{V}Z_{V}$ and the R-CS represented by $O_{R}X_{R}Y_{R}\;Z_{R}$.

The transformation matrix TRG in Figure 5 describes the position and the orientation of the R-CS with respect to the G-CS. Similarly, the identification matrix TVR describes the position and orientation of the V-CS with respect to the R-CS, which can be denoted by the *X-Y-Z* fixed angles of the R-CS. Concretely, set the R-CS fixed, the V-CS rotates γ along the *X_R_*-axis, then rotates *β* along the *Z_R_*-axis, and rotates α along the *Y_R_*-axis, at last translates *q_x_*,*q_y_*,*q_z_* along the *X_R_*-axis, *Y_R_*-axis, *Z_R_*-axis, respectively. After that we can get the current V-CS. Table 1 shows the identification parameters, and our goal is to determine the six identification parameters. The 3D coordinates of the terrain obtained by the vision sensor can be transferred to the R-CS by the transformation of the identification matrix TVR.

**Table 1:** The identification parameters.

Fixed Axes	Identification Angles	Identification Positions
***X_R_***	*γ*	*q_x_*
***Y_R_***	*α*	*q_y_*
***Z_R_***	*β*	*q_z_*

Equation (1) describes TVR in detail:

(1) TVR(α,β,γ,px,py,pz) =[RVRPVR01]=[t11t12t13t14t21t22t23t24t31t32t33t340001]

where:

(2) RVR=RYR(α)⋅RZR(β)⋅RXR(γ)=[cosα⋅cosβ−cosα⋅sinβ⋅cosγ+sinα⋅sinγcosα⋅sinβ⋅sinγ+sinα⋅cosγsinβcosβ⋅cosγ−cosβ⋅sinγ−sinα⋅cosβsinα⋅sinβ⋅cosγ+cosα⋅sinγ−sinα⋅sinβ⋅sinγ+cosα⋅cosγ]

(3) PVR=[pxpypz]T

## 4. Proposed Identification Methodology

Section 4 presents the novel identification model and method in detail.

**Figure 6:**
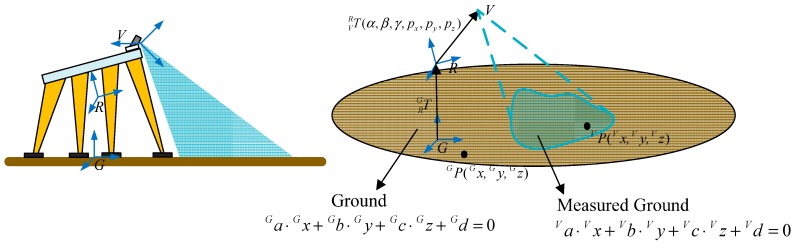
Modeling of the method.

As Figure 6 shows, P(x,y,z) is an arbitrary point on the ground plane, PV is with respect to the V-CS and PG is with respect to the G-CS. PV and PG fulfill Equation (4):

(4) PG= TRG ⋅TVR⋅PV

where TVR is the identification matrix we proposed in Section 3, and TRG is the transformation matrix from the R-CS to the G-CS. PV can be detected by the 3D vison system and fulfills a standard plane Equation (5):

(5) aV⋅xV+bV⋅yV+cV⋅zV+dV=0

where aV,bV and cV fulfill the formula aV2+bV2+cV2=1. The upper left mark V in Equation (5) denotes the variables are with respect to the V-CS PG, which is with respect to the G-CS, fulfills the following standard plane Equation (6):

(6) aG⋅xG+bG⋅yG+cG⋅zG+dG=0

where aG,bG and cG fulfill the formula aG2+bG2+cG2=1. The upper left mark G in Equation (6) denotes the variables are with respect to the G-CS. In our work, the ground fulfills the following plane Equation (7):

(7) yG=0

The term TVR can be computed by solving the constraint Equation (4). TRG, representing the relationship between the robot and the ground, can be obtained using the model presented in Section 4.2. In our methodology, TVR is not computed by recognizing some certain points *P*. Instead, we estimate the ground plane from the point cloud detected by the 3D vision system. Then we have developed an algorithm which will be presented in Section 4.3 that formulates the identification problem as an optimization problem. The above modeling can reduce recognition errors and avoid measurement errors.

### 4.1. Estimation of the Ground Plane

**Figure 7:**
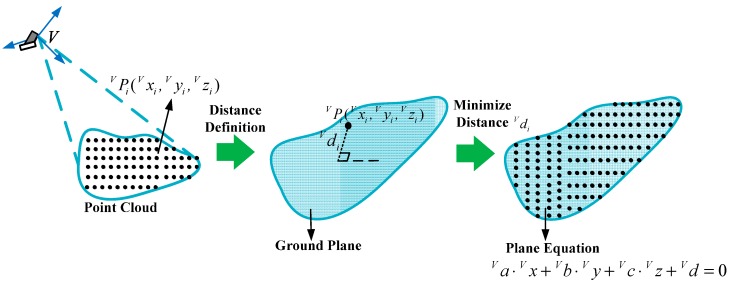
Estimation approach of the ground plane.

diV in Equation (8) is the distance from the detected point to the ground plane, as Figure 7 shows:

(8) diV=|aV⋅xiV+bV⋅yiV+cV⋅ziV−dV|

Most detected points belong to the ground plane, and diV should be 0. So the planar parameters aV,  bV,  cV,  dV can be computed by minimizing the value of diV:

(9) ε=∑i=1ndiV2=∑i=1n(aV⋅xiV+bV⋅yiV+cV⋅ziV−dV)2

ε in Equation (9) is defined to facilitate the computation. The Lagrange multiplier method is used to find the minimum value of ε. The Lagrange function is given by:

(10) L(aV,bV,cV,dV)=ε+λ(a2V+b2V+c2V−1)

The following formula exists:

(11) ∂L∂(dV)=−2∑i=1n(aV⋅xiV+bV⋅yiV+cV⋅ziV−dV)=0

Equation (12) can be obtained from Equation (11):

(12) dV=aV⋅∑i=1nxiVn+bV⋅∑i=1nyiVn+cV⋅∑i=1nziVn=aV⋅x¯V+bV⋅y¯V+cV⋅z¯V

Substituting Equation (12) into Equation (8), we can obtain the Equation (13):

(13) diV=|aV⋅(xiV−x¯V)x¯V+bV⋅(yiV−y¯V)+cV⋅(ziV−z¯V)|

There also exist the following equations:

(14) ∂L∂(aV)=2∑i=1n(aV⋅ΔxiV+bV⋅ΔyiV+cV⋅ΔziV)⋅ΔxiV+2λ⋅aV=0∂L∂(bV)=2∑i=1n(aV⋅ΔxiV+bV⋅ΔyiV+cV⋅ΔziV)⋅ΔyiV+2λ⋅bV=0∂L∂(cV)=2∑i=1n(aV⋅ΔxiV+bV⋅ΔyiV+cV⋅ΔziV)⋅ΔziV+2λ⋅cV=0

Equation (14) can be rewritten as a matrix equation:

(15) A⋅[aVbVcV]=[∑i=1nΔxiV⋅ΔxiV∑i=1nΔyiV⋅ΔxiV∑i=1nΔziV⋅ΔxiV∑i=1nΔxiV⋅ΔyiV∑i=1nΔyiV⋅ΔyiV∑i=1nΔziV⋅ΔyiV∑i=1nΔxiV⋅ΔziV∑i=1nΔyiV⋅ΔziV∑i=1nΔziV⋅ΔziV]⋅[aVbVcV]=−λ⋅[aVbVcV]

Observing Equation (15), we can find that [aV,  bV,  cV]T is the eigenvector of matrix A and −λ is the corresponding eigenvalue, so aV,  bV,  cV can be computed using the matrix eigenvector calculation method. Generally, matrix A has three eigenvalues and three different groups of eigenvectors correspondingly. diV can be obtained from Equation (13). The set of aV,  bV,  cV minimizing ε is the right set. After that, dV can be computed from Equation (12).

Because detection errors and influences of the outer environment exist in the identification process, some abnormal points have large errors, and some other points do not belong to the ground plane. These two kinds of points are called bad points, and a statistical method is used to exclude the bad points. Bad points can be removed, when the distances from them to the ground plane are larger than the standard value.

**Figure 8:**
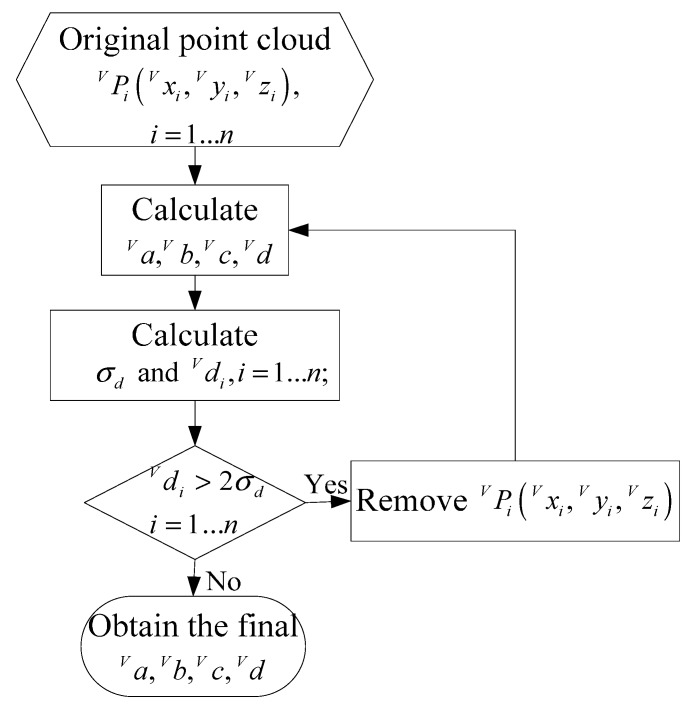
The flow chart of the ground plane estimation.

Figure 8 describes the estimation process of the ground plane. First, aV,  bV,  cV,  dV are computed using all the point cloud, and diV can be calculated from Equation (13), respectively. Then the standard deviation $\sigma_{d}$ of diV is obtained from Equation (16). Bad points are removed by comparing diV with $2\sigma_{d}$, the planar parameters are computed using the remaining point cloud again. We do so repeatedly until all the values of diV are less than $2\sigma_{d}$, and the final aV,  bV,  cV,  dV can be obtained. We have verified the estimation approach by simulations and experiments, the results show that the approach has well robustness and high precision:

(16) σd=∑i=1n(diV−d¯V)2(n−1)

where d¯V=∑i=1ndiVn.

### 4.2. Relationship Model between the Legged Robot and the Ground

In this section, the relationship model between the legged robot and the ground is established to accurately compute the robot’s position and orientation (denoted by TRG) with respect to the G-CS. The detailed expression of TRG is shown in Equation (17), whose formation process is similar to TVR. $\gamma^{\prime },\beta^{\prime },\alpha^{\prime }$ are angles that the robot rotates along the $X_{G}\text{-axix},Z_{G}\text{-axis and }Y_{G}\text{-axis}$ successively with respect to the fixed G-CS, and ${p_{x}}^{\prime },{p_{y}}^{\prime },{p_{z}}^{\prime }$ are distances that the robot translates along the $X_{G}\text{-axix},Y_{G}\text{-axis}$ and $Z_{G}\text{-axis}$ respectively. RRG is the orientation matrix, and PRG is the translation vector:

(17) TRG(α′,β′,γ′,px′,py′,pz′)=[RRGPRG01]=[a11a12a13a14a21a22a23a24a31a32a33a340001]

where:

(18) RRG=RYG(α′)⋅RZG(β′)⋅RXG(γ′)=[cosα′⋅cosβ′−cosα′⋅sinβ′⋅cosγ′+sinα′⋅sinγ′cosα′⋅sinβ′⋅sinγ′+sinα′⋅cosγ′sinβ′cosβ′⋅cosγ′−cosβ′⋅sinγ′−sinα′⋅cosβ′sinα′⋅sinβ′⋅cosγ′+cosα′⋅sinγ′−sinα′⋅sinβ′⋅sinγ′+cosα′⋅cosγ′]

(19) PRG=[px′py′pz′]T

**Figure 9:**
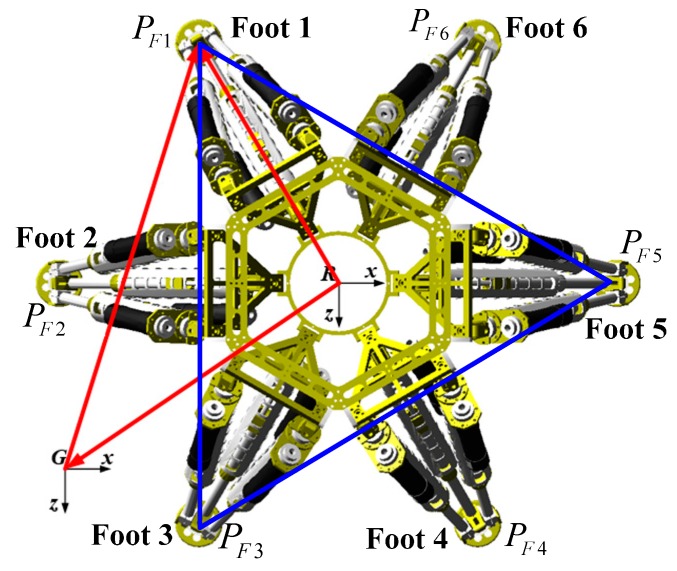
Initial state of the robot.

The initial position and orientation of the robot is shown in Figure 9, $P_{F1},P_{F2},P_{F3},P_{F4},P_{F5},P_{F6}$ denote positions of its feet. The positions of actuation joints are needed to be solved in order to set the robot to a known pose matrix TRG.

There exists Equation (20):

(20) PF1R=T-1RGPF1G

where PF1R is the position of Foot 1 with respect to the R-CS, and PF1G is a known position of Foot 1 with respect to the G-CS. Similarly, other feet have the same equations, we can obtain PF2R,PF3R,PF4R,PF5R,PF6R too. Then the positions of actuation joints can be obtained by the robot inverse kinematics.

The robot can reach the set pose if the actuation joints are driven to the calculated positions. The important point here is that there may be deviations between the real pose and the set pose because of the manufacture and installation errors. However, it is quite important to reduce errors during the whole identification process in order to increase the identification precision. Therefore, the real pose is calculated by the following derivations.

Similarly, PF1G,PF2G,PF3G,PF4G,PF5G,PF6G are known. Foots 1, 3 and 5 are chosen to calculate the real pose. As Figure 9 shows, there exist the following relations:

(21) RPF1→G=RG→G+GPF1→GRPF3→G=RG→G+GPF3→GRPF5→G=RG→G+GPF5→G

where the upper left mark *G* represents all the geometric relations are built with respect to the G-CS. Equation (22) can be obtained from Equation (21):

(22) |RPF1→G|2=(x1G−px′)2+(y1G−py′)2+(z1G−pz′)2|RPF3→G|2=(x3G−px′)2+(y3G−py′)2+(z3G−pz′)2|RPF5→G|2=(x5G−px′)2+(y5G−py′)2+(z5G−pz′)2

where (x1G,y1G,z1G) denotes the coordinates of Foot 1 with respect to the G-CS. Equation (23) can be derived based on the robot forward kinematics:

(23) |RPF1→R|2=x1R2+y1R2+z1R2|RPF3→R|2=x3R2+y3R2+z3R2|RPF5→R|2=x5R2+y5R2+z5R2

where (x1R,y1R,z1R) denotes the coordinates of Foot 1 with respect to the R-CS. The norms of vectors $\vec{RP_{F1}},\vec{RP_{F3}},\vec{RP_{F5}}$ are constant, so the following equations can be obtained:

(24) x1R2+y1R2+z1R2=(x1G−px′)2+(y1G−py′)2+(z1G−pz′)2x3R2+y3R2+z3R2=(x3G−px′)2+(y3G−py′)2+(z3G−pz′)2x5R2+y5R2+z5R2=(x5G−px′)2+(y5G−py′)2+(z5G−pz′)2

By solving the above equations, the real translation vector PRG are calculated. Additionally, there exists Equation (25):

(25) RRG⋅[RPF1→R,RPF3→R,RPF5→R]=[GPF1→G−PRG,GPF3→G−PRG,GPF5→G−PRG]

From Equation (25), the real orientation matrix RRG is computed too. Thus, the real translation matrix TRG can be calculated from Equations (24) and (25).

### 4.3. Formulation of the Identification Function

The following equations are obtained from Equation (4):

(26) xG=(a11⋅t11+a12⋅t21+a13⋅t31)⋅xV+(a11⋅t12+a12⋅t22+a13⋅t32)⋅yV+(a11⋅t13+a12⋅t23+a13⋅t33)⋅zV+a11⋅t14+a12⋅t24+a13⋅t34+a14yG=(a21⋅t11+a22⋅t21+a23⋅t31)⋅xV+(a21⋅t12+a22⋅t22+a23⋅t32)⋅yV+(a21⋅t13+a22⋅t23+a23⋅t33)⋅zV +a21⋅t14+a22⋅t24+a23⋅t34+a24zG=(a31⋅t11+a32⋅t21+a33⋅t31)⋅xV+(a31⋅t12+a32⋅t22+a33⋅t32)⋅yV+(a31⋅t13+a32⋅t23+a33⋅t33)⋅zV+a31⋅t14+a32⋅t24+a33⋅t34+a34

Because PG(xG,yG,zG) fulfills Equation (7), the following Equation is obtained from the second expression of Equation (26):

(27) (a21⋅t11+a22⋅t21+a23⋅t31)⋅xV+(a21⋅t12+a22⋅t22+a23⋅t32)⋅yV  +(a21⋅t13+a22⋅t23+a23⋅t33)⋅zV  +a21⋅t14+a22⋅t24+a23⋅t34+a24=0

**Figure 10:**
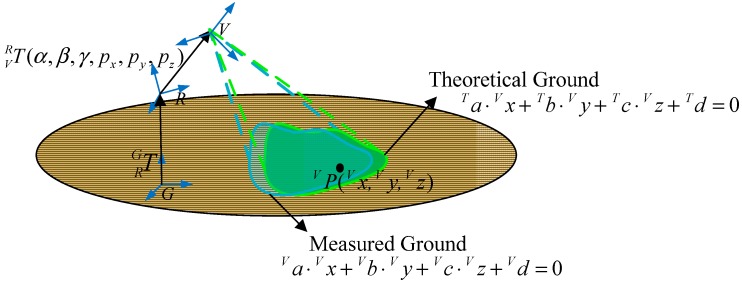
Formulation of the identification function.

As Figure 10 shows, Equation (27) is the theoretical ground equation with respect to the V-CS. aT,bT,cT,dT in Formula (28) represent theoretical ground planar parameters:

(28) aT=a21⋅t11+a22⋅t21+a23⋅t31bT=a21⋅t12+a22⋅t22+a23⋅t32cT=a21⋅t13+a22⋅t23+a23⋅t33dT=a21⋅t14+a22⋅t24+a23⋅t34+a24

Through derivations, we find there exists Equation (29):

(29) $$\begin{array}{l} {\left(a_{21}\cdot t_{11}+a_{22}\cdot t_{21}+a_{23}\cdot t_{31}\right)}^{2}+{\left(a_{21}\cdot t_{12}+a_{22}\cdot t_{22}+a_{23}\cdot t_{32}\right)}^{2}\\ +{\left(a_{21}\cdot t_{13}+a_{22}\cdot t_{23}+a_{23}\cdot t_{33}\right)}^{2}\text{=1} \end{array}$$

Theoretically, the measured ground coincides with the theoretical ground as shown in Figure 10. Because of Equation (29), Equation (27) is a standard plane equation, so it is obvious that Equation (27) is the same as Equation (5) derived in Section 4.1. Then the following four equations can be obtained:

(30) a21⋅t11+a22⋅t21+a23⋅t31=aVa21⋅t12+a22⋅t22+a23⋅t32=bVa21⋅t13+a22⋅t23+a23⋅t33=cVa21⋅t14+a22⋅t24+a23⋅t34+a24=dV

where $t_{11},\;t_{12},\;t_{13},\;t_{14},\;t_{21},\;t_{22},\;t_{23},\;t_{24},\;t_{31},\;t_{32},\;t_{33},\;t_{34}$ are variables associated with $\alpha ,\beta ,\gamma ,p_{x},p_{y},p_{z}$. A nonlinear function *F*, which consists of six identification parameters, can be defined as Equation (31):

(31) F=(a21cosα⋅cosβ+a22sinβ−a23sinαcosβ−aV)2+[a21(−cosα⋅sinβ⋅cosγ+sinα⋅sinγ)+a22cosβ⋅cosγ+a23(sinα⋅sinβ⋅cosγ+cosα⋅sinγ)−bV]2 +[a21(cosα⋅sinβ⋅sinγ+sinα⋅cosγ)−a22cosβ⋅sinγ+a23(−sinα⋅sinβ⋅sinγ+cosα⋅cosγ)−cV]2+(a21⋅px+a22⋅py+a23⋅pz+a24−dV)2

Generally, the legged robot has six DOFS, which can be used to simplify the identification process and increase the identification precision. At the beginning, the robot is located in an initial state, the $X_{R}$-axis and the $Z_{R}$-axis are parallel with the $X_{G}\text{-axis}$ and the $Z_{G}\text{-axis}$ respectively, the $Y_{R}\text{-axis}$ and the $Y_{G}\text{-axis}$ are collinear. Multiple groups of the robot poses and corresponding ground equations can be obtained by making the robot translate and rotate in space. Finally, identification parameters $\alpha ,\beta ,\gamma ,p_{x},p_{y},p_{z}$ can be obtained by minimizing the nonlinear function *F* using the LM algorithm.

## 5. Experimental Results and Discussion

In order to verify the proposed identification methodology, a series of experiments were carried out on the robot. The experimental results and related discussions are presented in this section.

**Figure 11:**
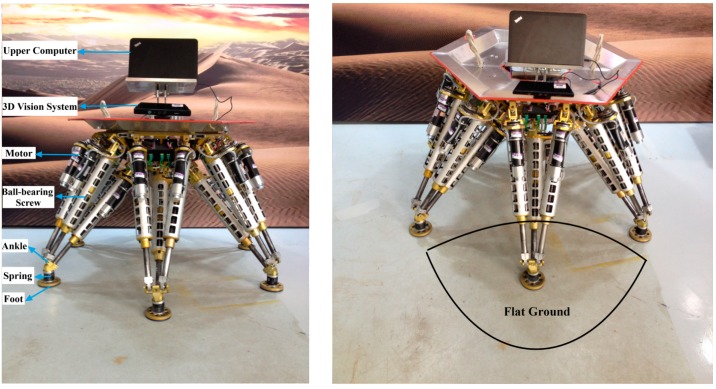
The experimental environment.

### 5.1. Set up and Identification Results

**Figure 12:**
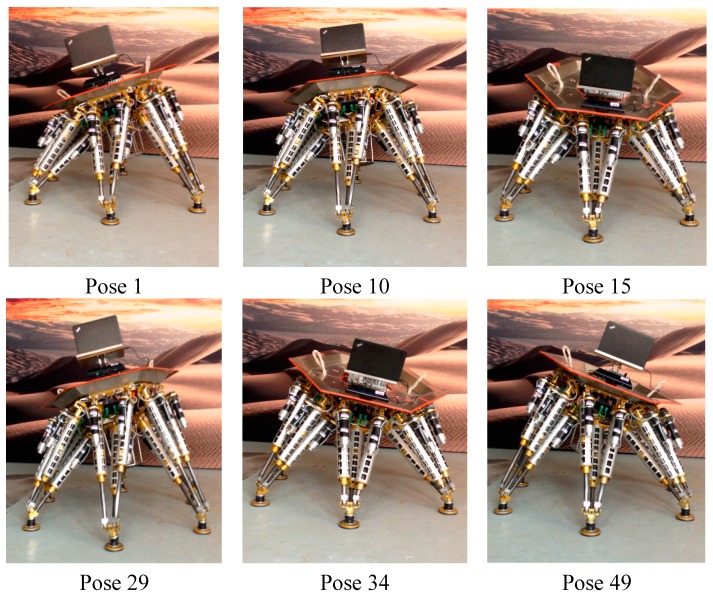
The robot poses.

Figure 11 shows the experimental environment, a small section of flat ground is in front of the robot. The 3D vision sensor is mounted at the top of the robot, and connected to the upper computer via USB. The 3D vision sensor is set to tilt down in order to guarantee that it can detect the ground. The upper computer controls the robot to reach 52 different groups of poses, and also controls the 3D vision sensor to detect the ground. When the robot reaches a set pose, the 3D vision sensor captures a depth image of the ground. Table A1 in the Appendix shows the 52 different groups of pose parameters, which are used in experiments. Taking into account the length of the paper, only six groups of the 52 experiments’ data are listed. But all the experimental data are discussed in detail. Figure 12 shows the six different groups of the robot poses.

**Figure 13:**
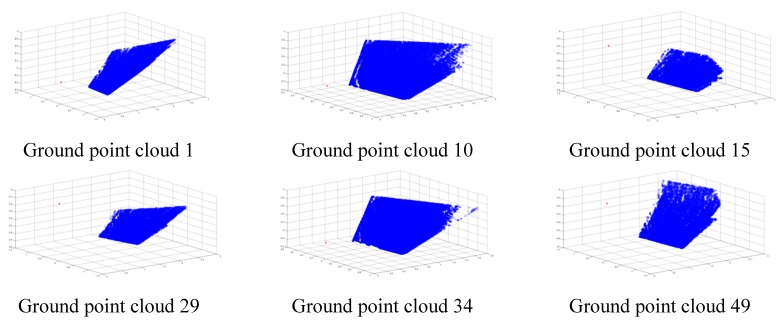
Ground point cloud.

Figure 13 shows the point cloud (blue points) of the ground corresponding to the above six groups of poses. The red point in Figure 13 denotes the origin of the 3D vision sensor. Some of cloud points having larger errors are removed using the approach proposed in Section 4.1, thus blue points far away from the 3D vision sensor are sparse. Correspondingly, Table 2 shows the six measured ground equations which are computed based on the approach in Section 4.1.

**Table 2:** Measured ground equations.

Number	Measured Ground Equation
**ground equation 1**	−0.5515xV+0.7802yV−0.2952zV+0.9916=0
**ground equation 10**	−0.7778xV+0.5839yV−0.2324zV+0.8372=0
**ground equation 15**	−0.3068xV+0.9504yV−0.0504zV+1.1626=0
**ground equation 29**	−0.3388xV+0.9312yV−0.1345zV+1.1656=0
**ground equation 34**	−0.8102xV+0.5668yV+0.1490zV+0.8378=0
**ground equation 49**	−0.4428xV+0.8957yV−0.0393zV+1.1721=0

After calculating the transformation matrices of the robot, identification parameters can be obtained using the algorithm in Section 4.3. The computed results are as shown by Equation (32):

(32) $$\begin{array}{l} \alpha =-0.4398{}^{\circ},\beta =-37.4072{}^{\circ},\gamma =-3.0471{}^{\circ},\\ p_{x}\text{=527.8885mm},p_{y}\text{=364.8007mm},p_{z}=-33.4003mm \end{array}$$

Equation (33) shows the detailed expression of TVR:

(33) TVR=[0.79430.60700.0246527.8885−0.60750.79320.0422364.80070.0061−0.04850.9988−33.40030001]

### 5.2. Errors Analysis

Substituting TVR into Equation (4), theoretical ground equations with respect to the V-CS can be obtained. Table 3 shows six detailed expressions of the theoretical ground.

**Table 3:** Theoretical ground equations.

Number	Measured Ground Equation
**ground equation 1**	−0.5449xV+0.7827yV−0.3009zV+0.9894=0
**ground equation 10**	−0.7783xV+0.5826yV+0.2341zV+0.8363=0
**ground equation 15**	−0.3132xV+0.9483yV−0.0510zV+1.1632=0
**ground equation 29**	−0.3391xV+0.9309yV−0.1354zV+1.1639=0
**ground equation 34**	−0.8081xV+0.5698yV+0.1493zV+0.8390=0
**ground equation 49**	−0.4430xV+0.8957yV−0.0395zV+1.1733=0

**Figure 14:**
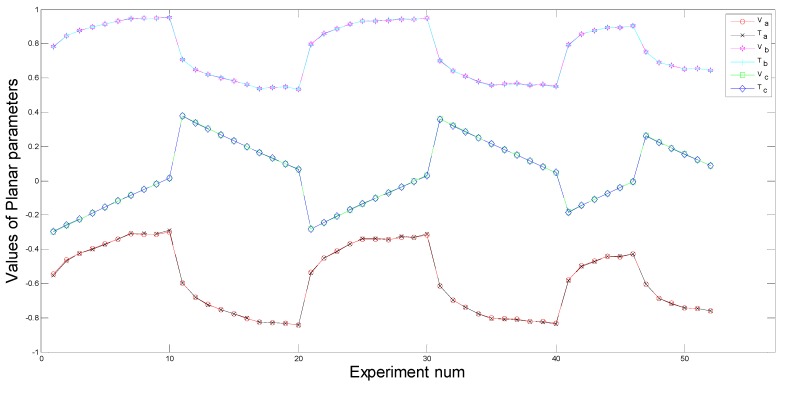
Measured angular parameters *vs.* theoretical angular parameters.

**Figure 15:**
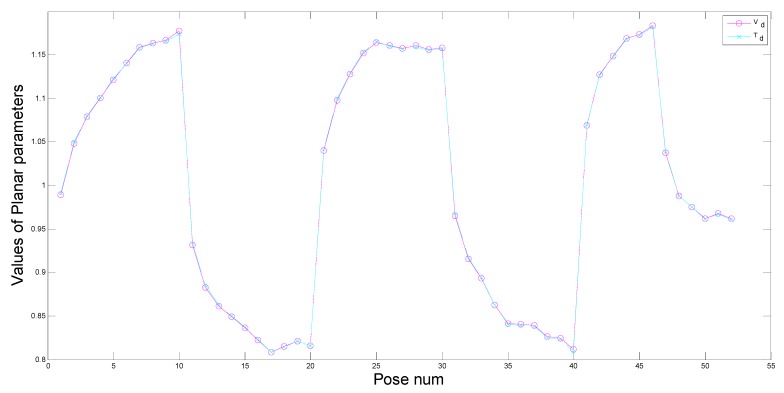
Measured distance *vs.* theoretical distance.

Figure 14 and Figure 15 illustrate measured ground planar parameters and theoretical ground planar parameters, respectively. From pose 1 to pose 10, pose 21 to pose 30, pose 41 to pose 46, the robot rotates along the positive direction. The rotation angle along the *Z*-axis and the translation distance increase gradually, while the rotation angle along the *X*-axis decreases gradually. From pose 11 to pose 20, pose 31 to pose 40, pose 47 to pose 52, the robot rotates along the negative direction. The rotation angle along the *Z*-axis and the translation distance increase gradually, while the rotation angle along the *X*-axis decreases gradually. Therefore, gradual increase and decrease of the planar parameters appear in Figure 14 and Figure 15 correspondingly. Values of theoretical planar parameters aT,bT,cT and measured planar parameters aV,bV,cV are shown in Figure 14, dT and dV are shown in Figure 15. Because a,b,c have the different geometric meaning from d, they are shown in different figures. From Figure 14 and Figure 15, we can clearly see that theoretical planar parameters and measured planar parameters have few errors and are nearly the same.

**Figure 16:**
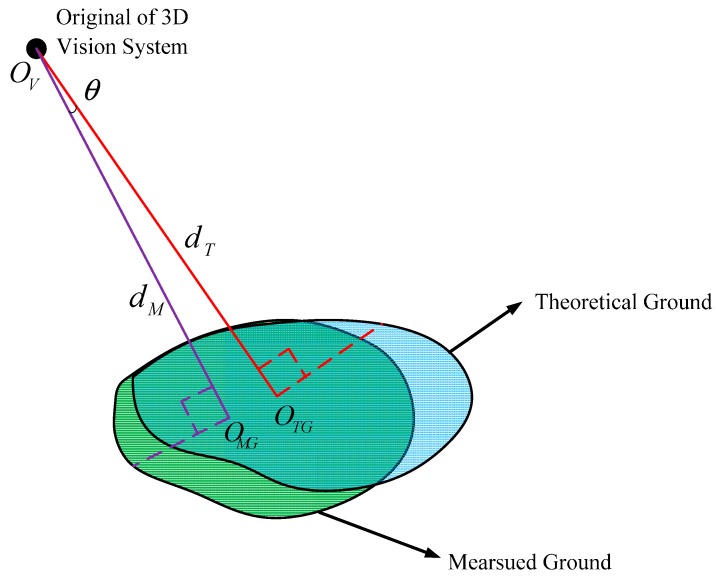
Error analysis.

In Figure 16, the blue region represents the theoretical ground, and the green region represents the ground measured by the 3D vision sensor. $O_{V}$ is the origin of the 3D vision sensor, $O_{V}O_{TG}$ is the normal of the theoretical ground, and $d_{T}$ is the distance from the origin to the theoretical ground. $O_{V}O_{MG}$ is the normal of the measured ground, and $d_{M}$ is the distance from the origin to the measured ground. θ denotes the angle between the theoretical ground and the measured ground. $e_{d}=\left|d_{T}-d_{M}\right|$ denotes the distance deviation between $d_{T}$ and $d_{M}$. In reality, the detected terrain is composed of many different planes, the index θ and $e_{d}$ can be used to describe the measurement precision.

**Figure 17:**
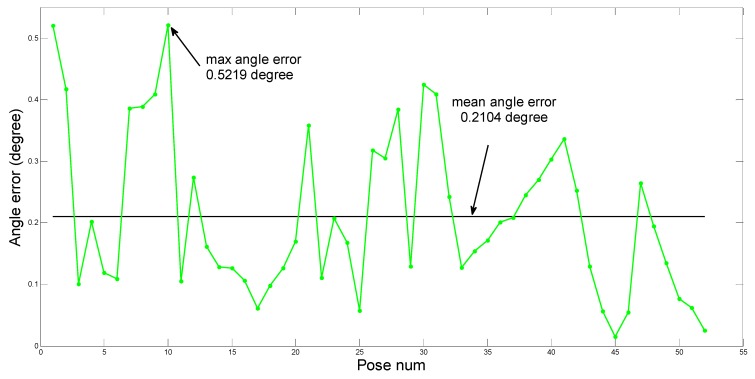
The angle error.

**Figure 18:**
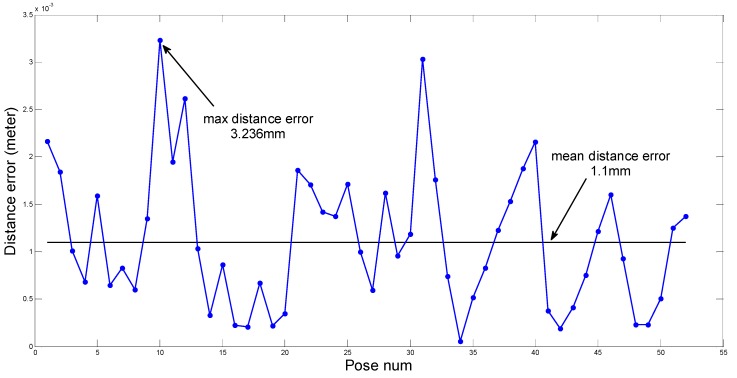
The distance error.

We have computed 52 groups of θ and $e_{d}$ corresponding to the set poses. The results are shown in Figure 17 and Figure 18, Figure 17 describes the value of θ and Figure 18 describes the value of $e_{d}$.

The mean value and the maximum value of θ and $e_{d}$ are marked in Figure 17 and Figure 18 separately. The mean value of θ is 0.2104°, and the maximum value is 0.5219°. The mean value of $e_{d}$ is 1.1 mm and the maximum value is about 3.236 mm. The robot’s minimum step height is 50 mm when it is walking, and its foot can rotate from −35° to 35° with respect to its leg. Thus the robot can bear the maximum angle error of 0.5219° and the maximum distance error of 3.236 mm easily. Above analysis results show that the identification precision fulfills the requirement of the robot, which validates our theory.

### 5.3. Robust Tests

**Figure 19:**
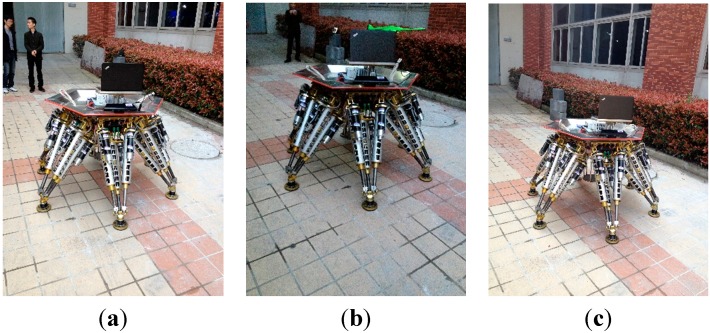
Robust test under different illumination conditions. (**a**) Normal illumination; (**b**) Weak illumination; (**c**) Strong illumination.

In this section, the robustness of the methodology is tested by carrying out identification experiments under two typical situations: different illumination conditions and different ground conditions. For the robust tests under different illumination conditions, the experiments are carried out at different times in an urban environment. As Figure 19 shows, the first experiment is carried out under normal illumination set at 4 pm as a reference, the second experiment in a dark set at 6 pm, and the third experiment in a bright set at 2 pm.

**Figure 20:**
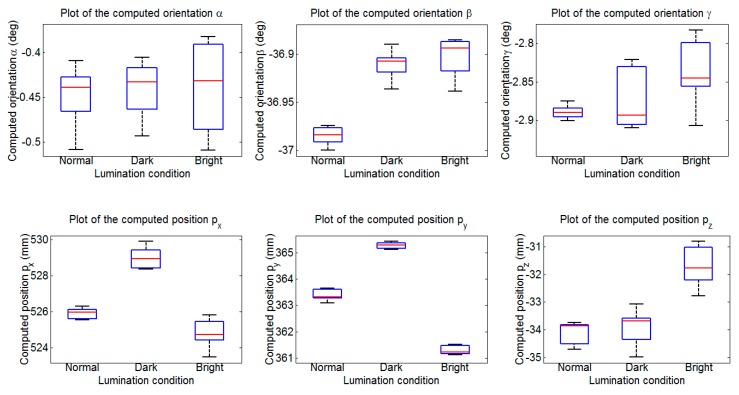
Box diagram of the identification results under different illumination conditions.

The experiment is executed 20 times under each illumination condition. We provide the mean and standard deviation of the identification results in Table 4 along with box plots in Figure 20 to illustrate their spread. Figure 20 shows the spread of the identification parameters under different illumination conditions. The boxes span the 25th and 75th percentiles, with the media depicted by the central line in the box plot. The tails of the box plots represent the range. As Figure 20 shows, the computed results vary most in the bright set. Concretely, the angles α are within about 0.13°, β are within about 0.05°, γ are within 0.12°, and the positions $p_{x}$ and $p_{z}$ are both within about 2 mm, $p_{y}$ are within about 0.4 mm. The more precise results have been achieved in the normal and dark sets. Table 4 shows the statistical results of these tests. The mean values of α, β, γ under three lumination conditions are nearly the same, as the mean values of positions differ less than 4 mm. The standard deviation obtained in the bright set is the maximum, the standard deviation of α is less than 0.052°, the standard deviation of $p_{x}$ is less than 0.8 mm. Nevertheless, the standard deviations in the bright set are relatively small compared to the results of Hoepflinger [29].

**Table 4:** Identification results under different lumination conditions.

	Mean			Standard Deviation		
	Normal	Weak Lumination	Strong Lumination	Normal	Weak Lumination	Strong Lumination
α (deg)	−0.4492	−0.4401	−0.4363	0.0327	0.0317	0.0517
β (deg)	−36.9849	−36.9103	−36.9026	0.0093	0.0149	0.0204
γ (deg)	−2.8893	−2.8718	−2.8383	0.0084	0.0400	0.0431
$p_{x}$ (mm)	525.8881	528.9832	524.8196	0.2941	0.5935	0.7932
$p_{y}$ (mm)	363.4033	365.2710	361.3017	0.2089	0.1166	0.1651
$p_{z}$ (mm)	−34.0852	−33.8849	−31.6874	0.4269	0.6543	0.7295

**Figure 21:**
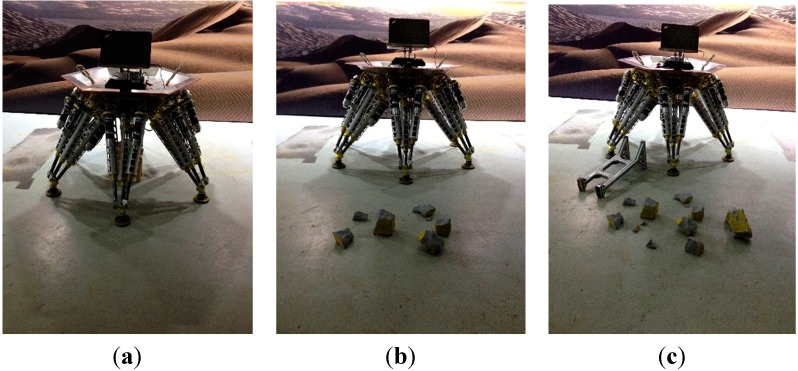
Robust test under different ground conditions. (**a**) Normal ground; (**b**) Little bumpiness; (**c**) Big bumpiness.

For the robustness test under different ground conditions, the experiments are performed on three different terrains. As Figure 21 shows, the first experiment is carried out on a flat ground as a reference, the second experiment on a slightly complex ground, and the third experiment on a considerably complex ground.

**Figure 22:**
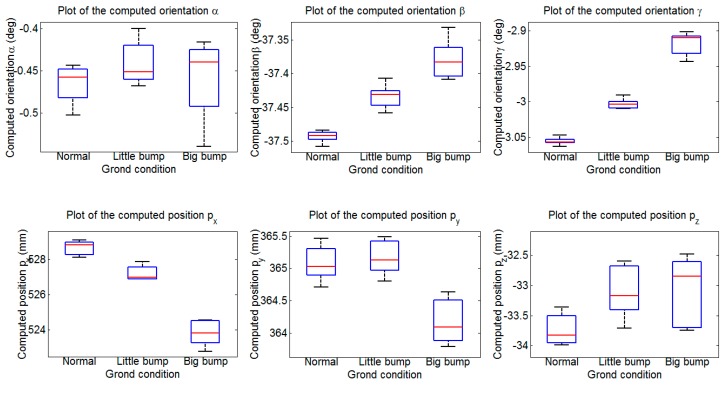
Box diagram of the identification results under different ground conditions.

The experiment is executed 20 times on each terrain. The mean and standard deviation of the identification results are provided in Table 5; and the results’ spread is illustrated with box plots in Figure 22. As Figure 22 shows; the identification precision on the flat ground is the highest. The most imprecise results are obtained on the considerably complex ground; the angles α are within about 0.124°; β are within about 0.076°; γ are within 0.414°; the positions $p_{x}$ are within 1.77 mm; $p_{y}$ are within 0.85 mm; $p_{z}$ are within 1.26 mm. The statistical results of the test are shown in Table 5. The mean values of α; β; γ obtained under three terrains are close to each other. The mean values of positions $p_{y}$ and $p_{z}$ obtained on the three terrains differ less than 1 mm; the mean value of position $p_{x}$ obtained on the considerably complex ground has about 5 mm difference compared to the ones obtained on the other two terrains. It can be observed that the standard deviations are sufficiently small; the maximum standard deviation of angle is less than 0.05° and the maximum standard deviation of position is less than 0.72 mm; both results being obtained on the considerably complex ground.

**Table 5:** Identification results under different ground conditions.

	Mean			Standard Deviation		
	Normal	Little Bumpiness	Big Bumpiness	Normal	Little Bumpiness	Big Bumpiness
α (deg)	−0.4657	−0.4425	−0.4592	0.0220	0.0264	0.0461
β (deg)	−37.4931	−37.4333	−37.3797	0.0079	0.0171	0.0278
γ (deg)	−3.0555	−3.0027	−2.9176	0.0048	0.0068	0.0161
$p_{x}$ (mm)	528.6361	527.2074	523.8542	0.3997	0.4042	0.7120
$p_{y}$ (mm)	365.0869	365.1528	364.1795	0.2708	0.2649	0.3441
$p_{z}$ (mm)	−33.7265	−33.0949	−33.0949	0.2574	0.4256	0.5747

To conclude this section, above box plots show how the illumination conditions and the complexity of the ground affect the identification precision. The identification results obtained under different illumination conditions and different ground conditions do not differ greatly and the standard deviations are quite small, which shows our method is very robust and stable and can be applied in some complex environments.

## 6. Use Case

A use case, underlying the importance and applicability of the methodology in the legged robots field, is presented next. In reality, a legged robot is often used in an unknown environment to execute daunting tasks. With the help of a vision sensor, the robot has a good knowledge of the environment. What’s more, after computing the extrinsic parameters relating the vision sensor and the legged robot, an accurate relationship between the robot and the terrain can be obtained. Thus the automatic locomotion can be implemented to execute tasks.

**Figure 23:**
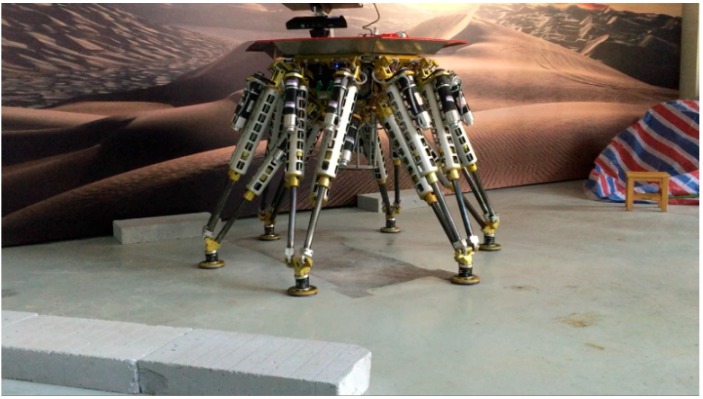
External view of the legged robot passing through obstacles.

As Figure 23 shows, the robot is in an unknown environment with obstacles. Based on the proposed methodology, the extrinsic parameters relating the sensor and the robot can be computed. The terrain map with respect to the robot can be built. Moreover, the accurate position and orientation of the obstacles are obtained from the terrain map. An automatic locomotion planning algorithm combining the terrain information is executed to plan the foot and body trajectories.

**Figure 24:**
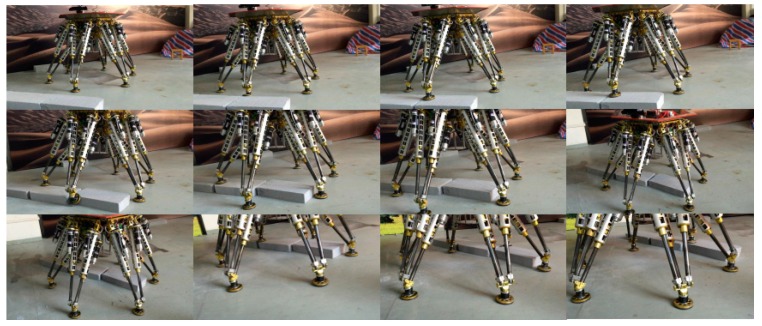
Snapshots of the legged robot passing through obstacles.

Figure 24 shows the whole process of passing through the obstacles, the robot body is regulated to move forward horizontally. During the whole process, the feet are placed at the planned footholds, so its body remains stable when walking on the obstacles. The results show a successful application of the methodology in the intelligent robotic field.

## 7. Conclusions

In this paper, we have presented a novel coordinate identification methodology for a 3D vision system mounted on a legged robot. Generally, the method can address the problem of extrinsic calibration between a 3D type vision sensor and legged robots, which few studies have worked on. The proposed method provides several advantages. Instead of using any kind of external tools (calibration targets and measurement equipment), our method only needs a small section of relatively flat ground, which can reduce recognition errors and avoid measurement errors. Moreover, the method needs no human intervention, and it is practical and easy to implement.

The theoretical contributions of this paper can be summarized as follows. An approach for estimating the ground plane is introduced based on optimization and statistical methods, and the relationship model between the robot and the ground is established too. The identification parameters are obtained from the identification function using the LM algorithm. Finally, a series of experiments are performed on a hexapod robot, and the identification parameters are computed using the proposed method. The calculated errors satisfy the requirements of the robot, which validates our theory. In addition, experiments in various environments are also performed, the results show that our methodology has good stability and robustness. A use case, in which the legged robot can pass through rough terrains after accurately obtaining the identification parameters, is also given to verify the practicability of the method. The work of this paper supplements relevant study in legged robots, and the method can be applied in a wide range of similar applications.

## Appendix

**Table A1:** The pose parameters of octopus.

Pose Number	$\alpha^{\prime }$ (°)	$\beta^{\prime }$ (°)	$\gamma^{\prime }$ (°)	${p_{x}}^{\prime }$ (mm)	${p_{y}}^{\prime }$ (mm)	${p_{z}}^{\prime }$ (mm)
**1**	0	2	20	0	615	0
**2**	0	8	18	0	619	0
**3**	0	11	16	0	623	0
**4**	0	13	14	0	627	0
**5**	0	15	12	0	631	0
**6**	0	17	10	0	635	0
**7**	0	19	8	0	639	0
**8**	0	19	6	0	643	0
**9**	0	19	4	0	647	0
**10**	0	20	2	0	651	0
**11**	0	−2	−20	0	617	0
**12**	0	−8	−18	0	621	0
**13**	0	−11	−16	0	625	0
**14**	0	−13	−14	0	629	0
**15**	0	−15	−12	0	633	0
**16**	0	−17	−10	0	637	0
**17**	0	−19	−8	0	641	0
**18**	0	−19	−6	0	645	0
**19**	0	−19	−4	0	649	0
**20**	0	−20	−2	0	653	0
**21**	0	3	19	0	690	0
**22**	0	9	17	0	694	0
**23**	0	12	15	0	698	0
**24**	0	15	13	0	698	0
**25**	0	17	11	0	694	0
**26**	0	17	9	0	690	0
**27**	0	17	7	0	686	0
**28**	0	18	5	0	682	0
**29**	0	18	3	0	678	0
**30**	0	19	1	0	674	0
**31**	0	−3	−19	0	692	0
**32**	0	−9	−17	0	696	0
**33**	0	−12	−15	0	700	0
**34**	0	−15	−13	0	696	0
**35**	0	−17	−11	0	692	0
**36**	0	−17	−9	0	688	0
**37**	0	−17	−7	0	684	0
**38**	0	−18	−5	0	680	0
**39**	0	−18	−3	0	676	0
**40**	0	−19	−1	0	672	0
**41**	0	1	13	0	730	0
**42**	0	7	11	0	734	0
**43**	0	9	9	0	738	0
**44**	0	11	7	0	742	0
**45**	0	11	5	0	746	0
**46**	0	12	3	0	749	0
**47**	0	−1	−13	0	732	0
**48**	0	−7	−11	0	736	0
**49**	0	−9	−9	0	740	0
**50**	0	−11	−7	0	744	0
**51**	0	−11	−5	0	748	0
**52**	0	−12	−3	0	750	0

## References

[1] Bazeille S., Barasuol V., Focchi M., Havoutis I., Frigerio M., Buchli J., Semini C., Caldwell D.G. Vision Enhanced Reactive Locomotion Control for Trotting on Rough Terrain. Proceedings of the 2013 IEEE International Conference on Technologies for Practical Robot Applications (TePRA).

[2] Walas K. Terrain Classification Using Vision, Depth and Tactile Perception. Proceedings of the 2013 RGB-D: Advanced Reasoning with Depth Cameras in Conjunction with RSS.

[3] Stelzer A., Hirschmuller H., Gorner M. (2012). Stereo-Vision-Based Navigation of a Six-Legged Walking Robot in Unknown Rough Terrain. Int. J. Robot. Res..

[4] Kolter J.Z., Kim Y., Ng A.Y. Stereo Vision and Terrain Modeling for Quadruped Robots. Proceeding of the IEEE International Conference on Robotics and Automation (ICRA ’09).

[5] Belter D., Skrzypczyński P. (2011). Rough Terrain Mapping and Classification for Foothold Selection in a Walking Robot. J. Field Robot..

[6] Ishigami G., Otsuki M., Kubota T. (2013). Range-Dependent Terrain Mapping and Multipath Planning Using Cylindrical Coordinates for a Planetary Exploration Rover. J. Field Robot..

[7] Kesper P., Grinke E., Hesse F., Wörgötter F., Manoonpong P. (2013). Obstacle/Gap Detection and Terrain Classification of Walking Robots Based on a 2D Laser Range Finder. Chapter.

[8] Kang T.K., Lim M.T., Park G.T., Kim D.W. (2013). 3D Vision-Based Local Path Planning System of a Humanoid Robot for Obstacle Avoidance. J. Electr. Eng. Technol..

[9] Wong C.C., Hwang C.L., Huang K.H., Hu Y.Y., Cheng C.T. (2011). Design and Implementation of Vision-Based Fuzzy Obstacle Avoidance Method on Humanoid Robot. Int. J. Fuzzy Syst..

[10] Bogdan Rusu R., Sundaresan A., Morisset B., Hauser K., Agrawal M., Latombe J.C., Beetz M. (2009). Leaving Flatland: Efficient Real-Time Three-Dimensional Perception and Motion Planning. J. Field Robot..

[11] Herrera D., Kannala J., Heikkilä J. (2011). Accurate and Practical Calibration of a Depth and Color Camera Pair. Computer Analysis of Images and Patterns.

[12] Li G., Liu Y., Dong L., Cai X., Zhou D. An Algorithm for Extrinsic Parameters Calibration of a Camera and a Laser Range Finder Using Line Features. Proceedings of the IEEE/RSJ International Conference on Intelligent Robots and Systems (IROS 2007).

[13] Guo C.X., Mirzaei F.M., Roumeliotis S.I. An Analytical Least-Squares Solution to the Odometer-Camera Extrinsic Calibration Problem. Proceedings of the 2012 IEEE International Conference on Robotics and Automation (ICRA).

[14] Geiger A., Moosmann F., Car O., Schuster B. Automatic Camera and Range Sensor Calibration Using a Single Shot. Proceedings of the 2012 IEEE International Conference on Robotics and Automation (ICRA).

[15] Pandey G., McBride J.R., Savarese S., Eustice R. Automatic Targetless Extrinsic Calibration of a 3D Lidar and Camera by Maximizing Mutual Information. Proceedings of the Twenty-Six AAAI Conference on Artificial Intelligence.

[16] Zhang Q., Pless R. Extrinsic Calibration of a Camera and Laser Range Finder (Improves Camera Calibration). Proceedings of the 2004 IEEE/RSJ International Conference on Intelligent Robots and Systems (IROS 2004).

[17] Huang P.S., Hong W.B., Chien H.J., Chen C.Y. Extrinsic Calibration of a Multi-Beam LiDAR System with Improved Intrinsic Laser Parameters Using V-Shaped Planes and Infrared Images. Proceedings of the 2013 11th IEEE IVMSP Workshop.

[18] Fernández-Moral E., González-Jiménez J., Rives P., Arévalo V. Extrinsic Calibration of a Set of Range Cameras in 5 Seconds without Pattern. Proceedings of the 2014 IEEE/RSJ International. Conference on Intelligent Robots and Systems (IROS 2014).

[19] Kwak K., Huber D.F., Badino H., Kanade T. Extrinsic Calibration of a Single Line Scanning Lidar and a Camera. 2011 IEEE/RSJ International Conference on Intelligent Robots and Systems (IROS).

[20] Agrawal A. Extrinsic Camera Calibration without a Direct View Using Spherical Mirror. Proceedings of the 2013 IEEE International Conference on Computer Vision (ICCV).

[21] Lébraly P., Deymier C., Ait-Aider O., Royer E., Dhome M. Flexible Extrinsic Calibration of Non-Overlapping Cameras Using a Planar Mirror: Application to Vision-Based Robotics. Proceedings of the 2010 IEEE/RSJ International Conference on Intelligent Robots and Systems (IROS).

[22] Hesch J.A., Mourikis A.I., Roumeliotis S.I. (2009). Mirror-Based Extrinsic Camera Calibration. Algorithmic Foundation of Robotics VIII.

[23] Zhou L. (2014). A New Minimal Solution for the Extrinsic Calibration of a 2D LIDAR and a Camera Using Three Plane-Line Correspondences. IEEE Sens. J..

[24] Kellyt J., Matthies L.H., Sukhatme G. Simultaneous Mapping and Stereo Extrinsic Parameter Calibration Using GPS Measurements. Proceedings of the 2011 IEEE International Conference on Robotics and Automation (ICRA).

[25] Wang C.C. (1992). Extrinsic Calibration of a Vision Sensor Mounted on a Robot. IEEE Trans. Robot. Autom..

[26] Strobl K.H., Hirzinger G. Optimal Hand-Eye Calibration. Proceedings of the 2006 IEEE/RSJ International Conference on Intelligent Robots and Systems.

[27] Dornaika F., Horaud R. (1998). Simultaneous Robot-World and Hand-Eye Calibration. IEEE Trans. Robot. Autom..

[28] Wongwilai N., Niparnan N., Sudsang A. Calibration of an Eye-in-Hand System Using SoftKinetic DepthSense and Katana Robotic Arm. Proceedings of the 2014 11th International Conference on Electrical Engineering/Electronics, Computer, Telecommunications and Information Technology (ECTI-CON).

[29] Hoepflinger M.A., Remy D.C., Hutter M., Siegwart R.Y. Extrinsic RGB-D Camera Calibration for Legged Robots. Proceedings of the 14th International Conference on Climbing and Walking Robots and the Support Technologies for Mobile Machines (CLAWAR).

[30] Pan Y., Gao F. (2013). A New 6-Parallel-Legged Walking Robot for Drilling Holes on the Fuselage. J. Mech. Eng. Sci..

[31] Yang P., Gao F. (2013). Leg Kinematic Analysis and Prototype Experiments of Walking-Operating Multifunctional Hexapod Robot. J. Mech. Eng. Sci..

[32] Khoshelham K. Accuracy Analysis of Kinect Depth Data. Proceedings of the International Archives of the Photogrammetry, Remote Sensing and Spatial Information Sciences, ISPRS Calgary 2011 Workshop.

[33] Khoshelham K., Elberink S.O. (2012). Accuracy and Resolution of Kinect Depth Data for Indoor Mapping Applications. Sensors.

